# Anticipatory Postural Adjustments During Gait Initiation in Stroke Patients

**DOI:** 10.3389/fneur.2019.00352

**Published:** 2019-04-17

**Authors:** Arnaud Delafontaine, Thomas Vialleron, Tarek Hussein, Eric Yiou, Jean-Louis Honeine, Silvia Colnaghi

**Affiliations:** ^1^CIAMS, Université Paris-Sud, Université Paris-Saclay, Orsay, France; ^2^CIAMS, Université d'Orléans, Orléans, France; ^3^ENKRE, Saint-Maurice, France; ^4^VEDECOM Institut, Versailles, France; ^5^Department of Public Health, Experimental and Forensic Medicine, University of Pavia, Pavia, Italy; ^6^Laboratory of Neuro-otology and Neuro-ophthalmology, IRCCS Mondino Foundation, Pavia, Italy

**Keywords:** anticipatory postural adjustments, gait initiation, stroke, rehabilitation, balance

## Abstract

Prior to gait initiation (GI), anticipatory postural adjustments (GI-APA) are activated in order to reorganize posture, favorably for gait. In healthy subjects, the center of pressure (CoP) is displaced backward during GI-APA, bilaterally by reducing soleus activities and activating the tibialis anterior (TA) muscles, and laterally in the direction of the leading leg, by activating hip abductors. In post-stroke hemiparetic patients, TA, soleus and hip abductor activities are impaired on the paretic side. Reduction in non-affected triceps surae activity can also be observed. These may result in a decreased ability to execute GI-APA and to generate propulsion forces during step execution. A systematic review was conducted to provide an overview of the reorganization which occurs in GI-APA following stroke as well as of the most effective strategies for tailoring gait-rehabilitation to these patients. Sixteen articles were included, providing gait data from a total of 220 patients. Stroke patients show a decrease in the TA activity associated with difficulties in silencing soleus muscle activity of the paretic leg, a decreased CoP shift, lower propulsive anterior forces and a longer preparatory phase. Regarding possible gait-rehabilitation strategies, the selected studies show that initiating gait with the paretic leg provides poor balance. The use of the non-paretic as the leading leg can be a useful exercise to stimulate the paretic postural muscles.

## Introduction

Gait initiation (GI) is a functional task classically used in the literature to investigate balance control mechanisms in healthy and pathological individuals (1, 2). GI may be a challenging task for balance control in stroke patients because of their sensory and motor impairments (3–5). Better knowledge of the altered balance control mechanisms in these patients is important to allow clinicians to target rehabilitation programs directed at minimizing the risk of falling. However, to date there is no review involving the changes in balance control mechanisms during GI in stroke patients.

**Figure 1:**
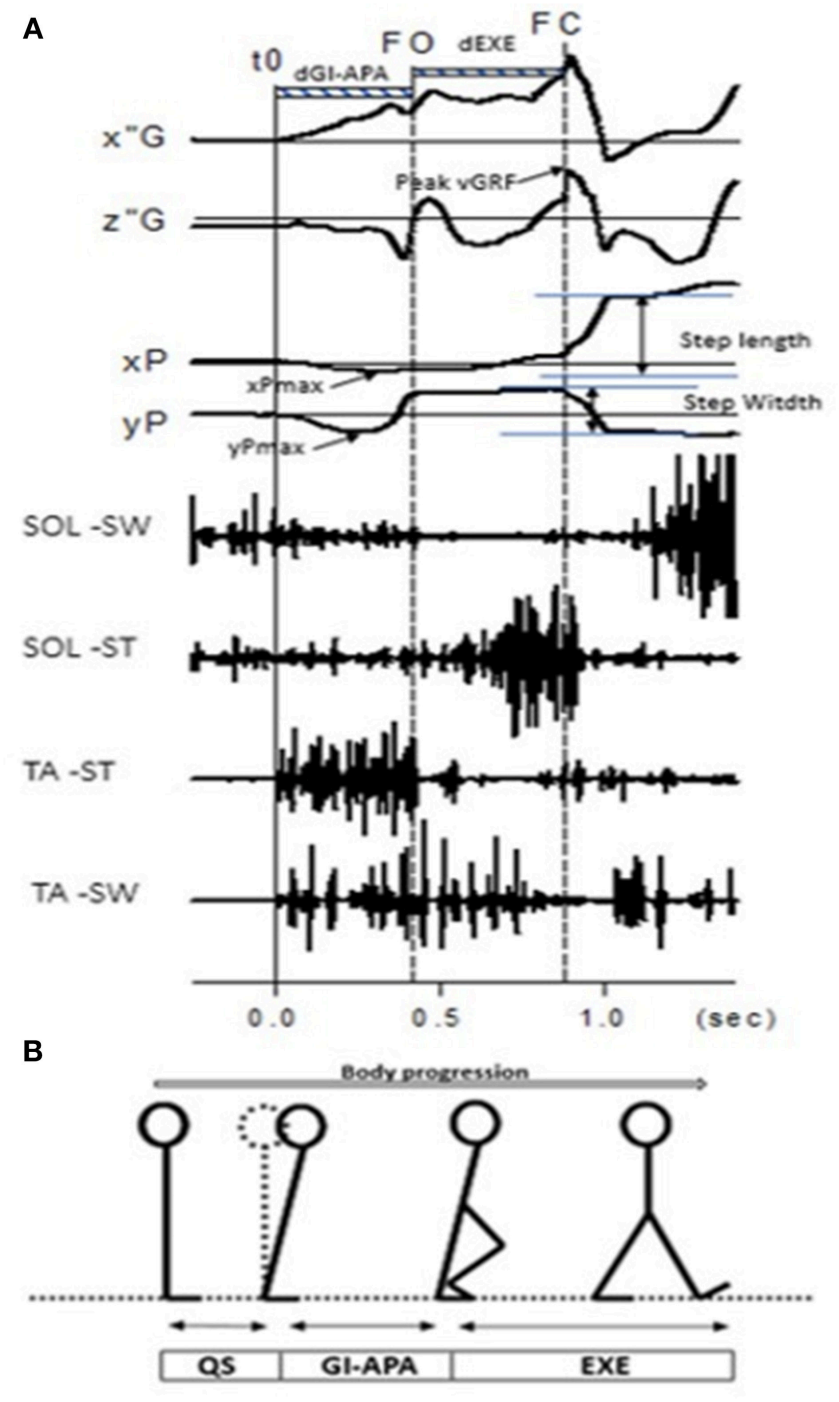
**(A)** Example of biomechanical traces obtained for one representative normal subject initiating gait at a spontaneous velocity (one trial). Anteroposterior direction x”G: anteroposterior center of mass velocity; z”G: vertical center of mass velocity; peak vGRF: the maximal moment of vertical Ground Reaction Force; xP, Center of pressure (COP) displacement; xPmax: maximal backward shift of CoP. Mediolateral direction yP, mediolateral COP displacement; yPmax, maximal mediolateral shift of COP. Vertical lines, t0 onset variation of biomechanical traces; FO, Swing foot off; FC, Swing foot contact. FL, Foot lift. Horizontal dashed line: dGI-APA, duration of Gait Initiation-Anticipatory Postural Adjustments; d-EXE, duration of execution phase. SOL-SW, Soleus electromyographical activity of swing leg; SOL-ST, Soleus electromyographical activity of stance leg; TA-ST, Tibialis Anterior electromyographical activity of stance leg; TA-SW, Tibialis Anterior electromyographical activity of swing leg. **(B)** Stick representation of the different phases and temporal events of gait initiation in a normal subject. QS, quiet standing; GI-APA, gait initiation-anticipatory postural adjustments; EXE, execution phase.

GI is composed of a postural phase (i.e., “anticipatory postural adjustments,” APA) where the dynamic phenomena necessary for a stable whole-body progression are generated, followed by the foot lift phase that ends at the time of swing toe clearance (i.e., body mass is transferred to the stance leg during this phase), and an execution phase that ends at the time of foot contact (2) (see Figures 1A,B).

Current literature describes gait initiation APA (GI-APA) components in the sagittal and frontal planes. In the sagittal plane, GI-APA are characterized by a backward center of pressure (CoP) shift with respect to the center of mass (CoM) position, providing the initial propulsive forces to progress forward (6, 7). This CoP shift is caused mainly by a bilateral reduction in soleus tonic activity and an increase in tibialis anterior (TA) tonic activity (8). In the frontal plane, the CoP is displaced toward the leading leg during GI-APA, which serves to move the CoM in the contralateral direction. This CoM shift helps disengage the leading leg for step execution and is crucial for maintaining stability (9). This mediolateral CoP shift is thought to be caused by leading leg abduction, accompanied by trailing leg ankle dorsiflexion and knee flexion (10). In post-stroke hemiparetic patients, damage to central structures results in a reorganization of GI-APA. The goal of this systematic review was to provide an overview of the characteristics of GI-APA in this population.

## Methods

### Methodology

The Preferred Reporting Items for Systematic Reviews and Meta-Analyses (PRISMA) methodology was employed for this systematic review. A bibliographical search was conducted in PubMed and Google Scholar using the following keywords: (“gait initiation” OR “step initiation”) AND (“stroke” OR “hemiparesis” OR “hemiplegia”). Only articles available in English or French and published before 31 August 2018 were selected. Additionally, manual searches were conducted from the reference list of each article found in the database until no further citations could be identified. To be included, the studies had to meet all of the following inclusion criteria: (1) anticipatory postural adjustments were explored during the paradigm of gait initiation with a force platform or electromyographical (EMG) activity (6, 8); for review see (2), (2) at least one stroke patent group was studied, (3) all types of studies (i.e., randomized and non-randomized control trials), (4) only articles in English or French, and (5) articles published in peer-reviewed journals.

GI-APA were retained as a main inclusion criteria because it plays a major role in postural stability and GI performance [for review see (2)], notably for stroke patients which are able to develop different motor strategies regarding the leg which initiates the gait [i.e., paretic or non-paretic leg; (3–5, 11, 12)].

On the contrary, studies were excluded if they observed gait without the study of anticipatory postural adjustments or if no stroke patients were included in the protocol.

### Study Selection

**Figure 2:**
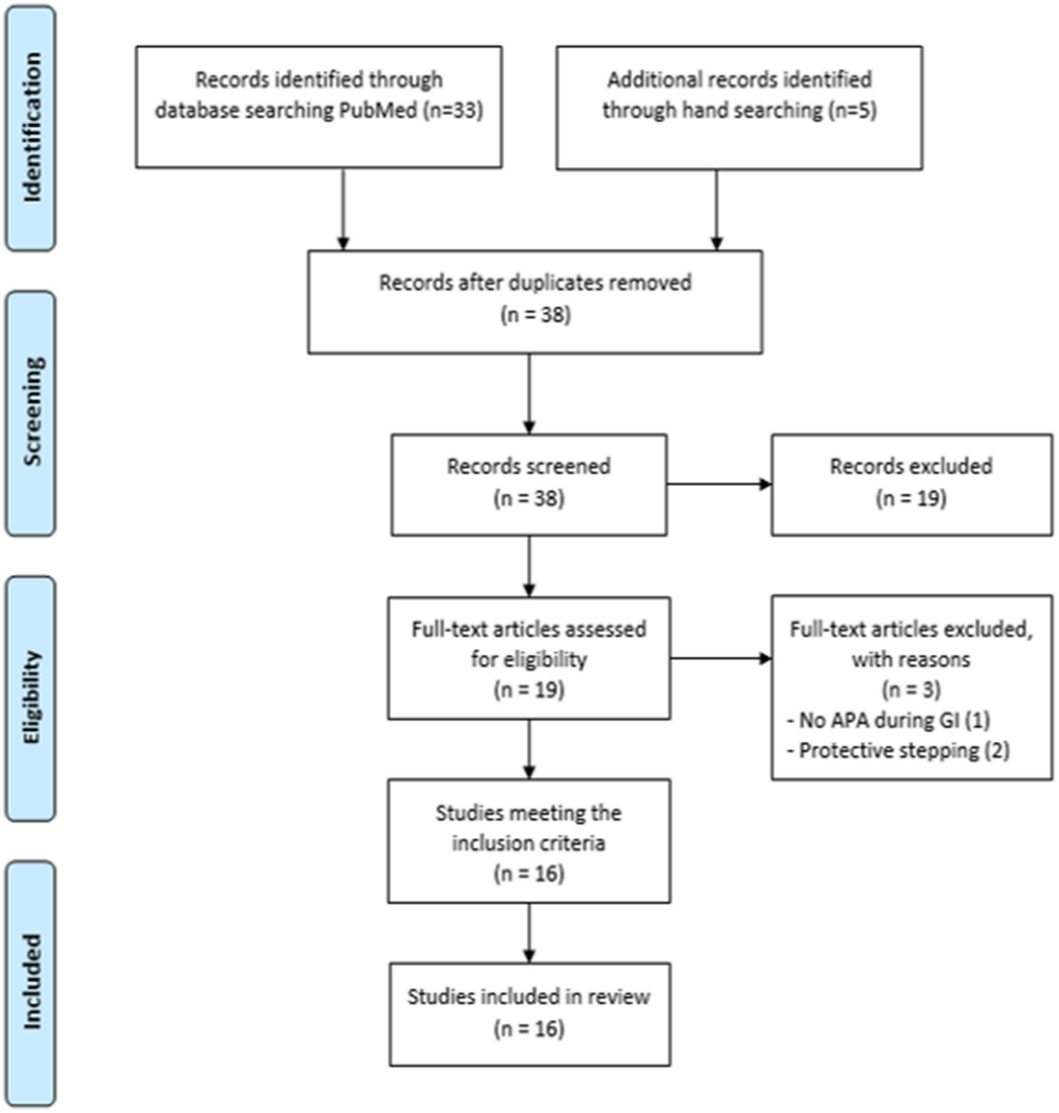
Study selection procedures and results reported in a flow-chart, as suggested by Moher et al. (15) and according to CONSORT statement.

Study selection was conducted in three steps (13, 14). First, two reviewers independently applied the inclusion and exclusion criteria to all the citations. Then, abstracts were screened to identify the titles representing a “best fit” with the aim of the present study. Finally, the reviewers read the full articles to reach a final decision on whether they should be included in the systematic review. When the relevance of a study was unclear from the abstract, the full article was read, and disagreements were resolved through group discussions with a third expert until a mutual consensus was reached. The reviewers met at the beginning, midpoint and final stages of the review process to discuss challenges and uncertainties related to study selection, and to go back and refine the search strategy if needed (Figure 2).

### Quality Assessment

The Downs and Black scale was used to assess the risk of bias and the methodological quality in the selected studies. The scale was used for its ability to provide an overview of the study quality of not only randomized controlled trials but also non-randomized studies (16). Additionally, this tool has been demonstrated to have a significant correlation with the valid PEDro scale (17, 18). The Downs and Black scale are divided into 27 questions and the answer to each can be “yes” (one point), “no” or “unable to determine” (zero point). Only the last item was modified from the original scale, as described previously by Trac et al. (16). The maximum score is 28: one point for each question, except for question five (two points).

After all of the articles were assessed and total quality scores were calculated, the level of evidence appraisal was conducted using Grades of Recommendation, Assessment, Development, and Evaluation-Confidence (GRADE) as recommended by The Cochrane Qualitative and Implementation Methods Group (19). The GRADE system classifies the quality of evidence in one of four levels: high, moderate, low, and very low. Evidence based on RCT begins as “high-quality,” whereas observational studies start with a “low quality” rating. Grading upwards may be warranted if the magnitude of the treatment effect is very large, if there is evidence of a dose-response relation or if all plausible biases would decrease the magnitude of an apparent treatment effect (20).

### Data Collection

Key items were charted from the selected articles by one of the authors. Item extraction was then checked by another author and disagreements were resolved by a third. The following data were noted for each selected article (Table 1):

- Author(s) and year of publication
- Study design
- Time elapsed between stroke onset and participation in the study
- Number of patients
- Mean age of patients
- Lesion sites
- Symptoms and signs (extent and severity)
- Gait initiation paradigm
- APA measurement methods (force platform and/or EMG)
- APA features.

**Table 1:** Clinical characteristics of the population in the selected studies.

**References**	**Post-stroke interval**	**Neurological lesion site**	**Symptoms**	**Symptoms severity**	**Treatment hints**
Gama et al. (21)	6 months	NS	Hemiparetic gait	NS	NS
Rajachandrakumar et al. (12)	43 days	NS	Hemiparesis	CMSA leg: 4.9 ± 1.3 CMSA foot: 4.6 ± 1.3	NS
Sharma et al. (22)	NS	NS	Hemiparesis	FMA: score NS	NS
Sousa et al. (23)	26 ± 11.3 months	Middle cerebral artery	Hemiparesis	FMA: score NS	NS
Sousa et al. (24)	24.9 ± 11.5 months	internal capsule	Hemiparesis	FMA: score NS	NS
Martinez et al. (25)	2.9 ± 1.1 yo	NS	Hemiparesis	NS	Use of stepping for balance rehabilitation
Ko et al. (26)	5.2 ± 3.1 yo	Cerebrum for 11 patients and brainstem for 1 patient	Hemiparesis	FMA: score NS	NS
Chang et al. (27)	NS	8 PMC lesions, 7 PMC spared	Hemiplegia	FMA motor part: 30.1 ± 2.5 for PMC group; 32.4 ± 1.1 for PMC spared group.	NS
Melzer et al. (28)	6.7 ± 4.1 yo	NS	Hemiparesis	BBS scale: 46 ± 4.5 for stroke patients	NS
Melzer et al. (29)	7.3 yo	Middle cerebral artery territory	Hemiparesis	BBS scale: 47.9 ± 6.1 on paretic side and 50.8 ± 4.3 on non-paretic side	NS
Bensoussan et al. (5)	7,66 yo (min 4, max 11)	Thalamic, internal capsule, sylvian area	Hemiplegia and equinus foot	FIM: 119–124 Barthel index: 95–100 Ashworth: 3–4	NS
Tokuno and Eng (30)	NS (i.e. minimum of 6 months post-stroke)	NS	Hemiparesis	CMSA: 8/14	Improve the propulsive force of the paretic leg when used as the trailing leg
Bensoussan et al. (4)	11 yo	Thalamic	Hemiplegia and equinus foot	Ashworth: 3/5 Barthel index: 95/100 FIM: 124–126 Functional ambulatory classification: 5/6	NS
Kirker et al. (31)	17 months	NS	Hemiparesis	NS	NS
Hesse et al. (11)	3.7 months	First ischemic stroke in middle cerebral artery	Hemiparesis	NS	NS
Brunt et al. (3)	11 weeks (min 4, max 23)	NS	Hemiplegia	NS	Improve symmetry in leg loading during quiet stance, or leg loading characteristics that are specific for gait initiation

*CMSA, Chedoke–McMaster Stroke Assessment; FMA, Fugl-Meyer motor Assessment; FIM, Functional Independence measure; NS, Non-Specified; PMC, PreMotor Cortex; vs., versus*.

## Results

### Selected Articles

Thirty-three articles emerged from the PubMed search. Five additional articles were identified through manual searching, for a total of 38 articles, 16 of which met the inclusion criteria (3–5, 11, 12, 21–23, 25–31). A total of 220 patients were included (Table 2).

**Table 2:** Methods and main results of the selected studies.

**Authors**	**Study design: (1) Main objectives (2) Study population (3) Outcomes (4) Methodology**	**Main results (only results related to GI-APA are reported)**
Gama et al. (21)	(1) First experiment: to compare gait initiation of individuals with stroke to age-matched non-disabled controls. Second experiment: to investigate how different amounts of partial body weight support (BWS) would influence the gait initiation of individuals with stroke. (2) 12 individuals with stroke (mean age 62 ± 4.6 years, 3 women and 9 men) and 12 non-disabled controls (mean age 62 ± 5years, 9 women, 3 men). (3) Initial weight loading on swing and stance limbs and ML distance between heels, displacement and velocity in ML and AP directions of the CoP trajectories, step length and velocity of swing limb. With a force plate (model 9286BA, Kistler) and a computerized gait analysis system (VICON, Inc.) with 7 infrared cameras. (4) First experiment: after a verbal command, the participants were instructed to walk barefoot toward the end of a 7-m walkway at a comfortable speed with no interruption. Individuals with stroke started walking with the paretic limb, and individuals without disabilities started walking with the right limb. Second experiment: same instructions but 3 different conditions of body weight unloading were employed (0, 15, and 30%) in a randomized order, only with stroke patients.	Experiment 1: individuals with stroke presented shorter ML and AP CoP displacements backward and toward the swing limb during the anticipatory phase. Individuals with stroke presented slower velocity of the CoP in both ML and AP directions during the anticipatory phase. Experiment 2: no effect of the partial body support on the anticipatory phase.
Rajachandrakumar et al. (12)	(1) To determine the prevalence, clinical correlates, and consequences of atypical GI-APA (absent and multiple GI-APA) post-stroke when initiating gait with the paretic and non-paretic legs. (2) 40 individuals with sub-acute stroke able to initiate gait without using a gait aid (mean age 67.8 years; 19 men, and 21 women). (3) Temporal and spatial characteristics of the GI-APA (ML COP and CoM shifts) with a force plate. (4) 6 trials of gait initiation. Participants were instructed to step with a specific leg prior to the trial, and to initiate forward gait at a self-selected speed when ready after a verbal “go” signal.	35% of all trials had atypical GI-APA and most participants had at least one trial with an atypical GI-APA. Prevalence of atypical GI-APA in individuals with stroke was significantly related to motor impairment. Absent or multiple GI-APA when starting with the non-paretic leg.
Sharma et al. (22)	(1) To characterize ground reaction forces (GRF) acting on the legs during GI after stroke to begin to build an understanding of how hemiparesis might affect GI. (2) 18 chronic stroke patients (9 men, 3 women; mean age 67.6 years, range 45–86). 9 patients left paretic and 9 patients right paretic. Twenty eight age similar healthy subjects (mean age 67.6, range 49–82).14 men and 14 women. (3) GRF was recorded from a Dual-Top Accusway force plate (AMTI, USA). (4) Subjects were instructed to initiate gait at the sound of an auditory cue. They self-selected their preferred speed. Left and right trials were collected in a random order and six trials were collected with each leg as the starting leg.	The legs of stroke patients generated lower anterior forces regardless of whether the trailing leg was paretic or non-paretic. Healthy subjects generated greater medial forces with their left leg than their right, but medial forces were equivalent for paretic and non-paretic legs. Patients with left hemisphere lesions generated greater lateral GRF with the non-paretic trailing leg than the paretic leg, and these non-paretic left leg lateral forces were also greater than the lateral forces generated by the left leg of healthy subjects. This left foot lateral GRF bias was not evident for patients with right hemisphere lesions.
Sousa et al. (23)	(1) To evaluate ankle GI-APA during gait initiation in chronic post-stroke subjects with lesion in the territory of middle cerebral artery (MCA). (2) 11 patients who had suffered a stroke at least 6 months earlier (mean age 53.10 years; 6 females, 5 males) and 12 healthy subjects (mean age 44.8 years; 5 females, 7 males). (3) Vertical, ML an AP ground reaction force with a force plate. The activity of Gastrocnemius Medialis (GM), Soleus (SOL) and Tibialis Anterior (TA) of both lower legs was assessed with EMG. (4) Subjects were asked to walk at self-adopted speed over a 5-m walkway, without explicit instructions. All participants from post-stroke group initiated gait with their contralesional leg.	Chronic post-stroke subjects present bilateral EMG SOL deactivation and lower tibialis anterior activity amplitude on the non- paretic leg and onset timing on both legs during gait initiation. This leads to decreased CoP displacement backward and toward the first swing leg.
Sousa et al. (24)	(1) To compare the reliability of CoP displacements during the postural phase of gait initiation calculated by two methods of detection the beginning of the postural phase in health and post-stroke participants. (2) 15 patients who had suffered a stroke at least 6 months earlier (8 females, 7 males) and 23 healthy participants (12 females, 11 males). (3) Vertical, ML and AP ground reaction force, as well as the values of moments of force in the frontal and sagittal planes, were acquired using a force plate (Bertec Corporation, USA). (4) To walk at self-selected speed over a 5 m walkway, without explicit instructions. Participants were asked to keep the starting leg consistent over all three trials.	Post-stroke participants present decreased CoP displacement during the postural phase of gait initiation and increased postural phase duration that impairs posture stability and motor performance.
Martinez et al. (25)	(1) To examine the stepping performance during voluntary and waist-pull perturbation-induced step initiation in people with chronic stroke. (2) 10 participants poststroke, (4 men and 6 women; mean age 59.6 years). Chronicity of stroke averaged 2.9 years, with 80% having right-sided weakness and ischemic stroke. (3) Ground reaction forces were recorded with 2 force platforms Body segment displacements were recorded using an optoelectronic system with 6-camera motion analysis system. (4) Voluntary and perturbation-induced stepping was studied consecutively. Three voluntary steps forward as soon and as fast as they could in response to a light “go” cue that followed a warning light stimulus by a variable delay period. Three trials for steps initiated with the non-paretic leg, 3 trials with the paretic leg. For the perturbation condition, steps were induced by forward postural perturbations.	GI-APA occurred in 100% of the voluntary stepping trials. The total postural phase duration was longer for voluntary stepping than induced straight forward stepping. The longer voluntary postural phase duration is primarily due to the prolonged unloading component. GI-APA amplitude was not different between conditions.
Ko et al. (26)	(1) To examine the immediate effect of natural (asymmetrical) and symmetrical weight bearing on the temporal events of ground reaction forces (GRF) and on timing and amplitude of lower distal muscle activity during GI in persons with hemiparesis. (2) 12 subjects (mean age 60.7 years; 7 males, 5 females) with unilateral hemiparesis. (3) EMG recording of Tibialis Anterior (TA) and Soleus (Sol) of both lower extremities. (4) The subject performed the following conditions of leg-loading distribution prior to executing a step in a randomized order: GI with the paretic leg during natural (asymmetrical) weight bearing; GI with the non-paretic leg during natural weight bearing; GI with the paretic leg during symmetrical weight bearing; and GI with the non-paretic leg during symmetrical weight bearing. Subjects began walking at their comfortable speed when they saw a visual cue (light). They were asked to initiate gait as quickly as possible after the visual cue. Subjects completed 5 trials.	Initiating gait with the non-paretic (leading) leg, the paretic TA muscle (trailing) was activated at the normal relative timing of percent GI cycle. When initiating gait with the non-paretic leg, the amplitude of the TA muscle on the paretic (trailing) leg was significantly increased about 27 to 36% compared with the paretic leg as leading leg in both natural and symmetrical leg- loading conditions. Improvement of standing symmetry during GI did not induce any adequate muscle activity on the impaired TA muscle for the effective weight shift necessary during GI. Delayed onset and less amplitude of TA activity when initiating with the paretic leg in both symmetrical and asymmetrical weight bearing conditions.
Chang et al. (27)	(1) To investigate whether premotor cortex (PMC) lesions influence stepping leg selection and stepping-related GI-APA in patients with a PMC lesion following a stroke. (2) 15 hemi-paretic patients (8 with PMC lesions, mean age 66.6 years and 7 PMC spared, mean age 65.1 years) and 8 age- and sex-matched healthy adults. (3) Vertical ground reaction force with 2 force plates EMG muscle activity from bilateral TA muscles was recorded. (4) All subjects were asked to prepare to react to an upcoming “go” signal (a green circle). Each subject underwent 6 right- and 6 left-leg-stepping trials in each condition. All subjects stepped independently without using any device.	The PMC lesions group exhibited the longest TA reaction time and contraction latency on both legs.
Melzer et al. (28)	(1) To explore differences in voluntary step behavior, a motor task of critical importance to prevent a fall from occurring, using the involved and uninvolved legs and to identify which of the step phases—step initiation-, preparation- or swing-phase—are markers for increased stepping time and thus, risk of falling. (2) 7 male and 3 female stroke survivors (61.7 ± 10 years old) 6.7 ± 4.1 years post- stroke and 10 healthy age-sex matched controls (61.7 ± 7.6 years old). (3) Center of pressure and ground reaction force data during step execution trials were collected and sampled at a frequency of 100 Hz, using a portable Kistler 9,287 force platform. (4) 12 step execution trials were performed in randomized order, 3 forward and 3 backward, for each leg, 6 stepping trials with the involved leg and 6 stepping trials with the uninvolved leg; healthy control subjects were instructed to step with their dominant leg.	The time to AP peak forces in the preparation and swing phases during single-task stepping were 80 and 162% longer, respectively, in the non-paretic leg compared with healthy controls. Also, the time to AP peak forces in the preparation and swing phases during single-task condition were 73 and 331% longer using the paretic leg in both stepping conditions compared with healthy subjects. AP peak force was significantly lower in both involved and uninvolved legs of stroke survivors compared with healthy controls, especially during the preparation phase in single- and dual-task conditions.
Melzer et al. (29)	(1) To ask whether the ability to quickly step—a motor task of critical importance to prevent a fall from occurring—was different in a group of chronic stroke survivors and healthy age- and sex-matched controls. (2) 16 hemiparetic subjects (age range, 46–77 year) who were 7.3 years poststroke, and 16 healthy controls matched for age and sex (age range, 43–81 years). (3) CoP and ground reaction force data during step execution trials were measured with a portable Kistler 9,287 force platform. (4) 3 forward and 3 backward stepping trials were performed by the uninvolved foot only in a randomized order for each of the 2 task conditions (single and dual task).	The preparatory phase durations in the chronic stroke survivors were significantly longer during the single task (93%), and only 35% longer in dual-task condition. A longer step initiation time was observed in stroke patients.
Bensoussan et al. (5)	(1) To assess the kinetic and kinematic characteristics of gait initiation during the various gait initiation phases in hemiplegic patients after stroke. (2) 3 hemiplegic patients after stroke (2 women, 1 man, 55, 35, and 21 years) were compared with 3 healthy subjects. (3) The kinematic study was carried out using an ELITE optoelectronic system (BTS spa, Milano, Italy) with 6 cameras The kinetic parameters were recorded via 2 AMTI force-plates (Advanced Mechanical Technology Inc., Watertown MA, USA). (4) The subject began walking at whatever speed he chose when instructed to do so by the operator. The foot to be used to initiate walking was chosen at random and specified by the operator by raising his left or right hand. Each subject performed 10 trials with each lower leg (20 trials/subjects).	The postural phase was longer when gait was initiated with the hemiplegic leg. The forces exerted by the affected leg were exerted backwards during the postural phase.
Tokuno and Eng (30)	(1) To determine the differences in gait initiation between a group of individuals with chronic stroke compared to a group of healthy adults. (2) 13 individuals with chronic stroke, 6 months post-stroke (8 males, 5 females, 59.3 years) and 13 healthy adults (8 males, 5 females, 64.7 years). (3) Gait initiation speed, duration of gait initiation, backward displacement of the center of pressure (CoP), initial loading of the legs during quiet stance, the peak vertical and antero-posterior (A-P) forces from the leading and trailing legs, and the A-P impulses from the leading and trailing leg with a force-plate. (4) Walking at their comfortable speed, without the use of any walking aid for a distance of five meters. The choice of leading leg was determined randomly by the experimenter. Three trials were collected from each lead leg condition.	Individuals with chronic stroke stood in a more asymmetrical manner. They relied only 42% on their paretic leg. The backward displacement of the CoP was smaller by a factor of approximately 2.5. The greater the ability an individual with stroke has to produce a larger peak A-P force and magnitude of A-P impulse with the paretic leg when used as the trailing leg, the faster the gait initiation speed they are able to achieve. Reduced propulsion by the paretic leg was observed.
Bensoussan et al. (4)	(1) To investigate the temporal, kinetic and kinematic asymmetry of gait initiation in one subject with hemiplegia with an equinus varus foot. (2)1 man with hemiplegia (21 years). (3) A kinetic analysis with two AMTI force plates and a kinematic analysis with an ELITE optoelectronic system. (4) The subject was asked to initiate gait at his preferred speed at a vocal signal. Operators randomly indicted to the subject which foot to use initiate gait. Ten trials for each foot	The distribution of body weight on the lower legs was asymmetrical. The forces exerted by the affected leg were exerted backwards during the postural phase. Reduced propulsion shortens monopodal phase and less body weight sharing by the paretic leg were observed.
Kirker et al. (31)	(1) To compare the pattern of pelvic girdle muscle activation in normal subjects and hemiparetic patients while stepping and maintaining standing balance. (2) 12 male and 5 female patients with stroke, with 13 right and 4 left hemiplegia's (mean age 54 years). Sixteen normal controls (mean age 46 years). (3) Surface EMGs of bilateral hip abductors (gluteus medius) and adductors (adductor longus), lateral torque component of the ground reaction forces, and pelvic displacement with a force-plate. (4) A sideways push task, a standing balance task and a gait initiation task. Forty trials were recorded, in alternate blocks of five trials leading with the left foot and five trials leading with the right foot.	The initial sideways weight shift is accompanied by increases in activity of the gluteus medius of the stepping leg and contralateral adductor. Lifting the weight off the leading leg is accompanied by large increases in activity of the trailing leg gluteus medius and leading leg adductor. Normal activation pattern of the hemiparetic leg muscles were observed.
Hesse et al. (11)	(1) To quantify a possible asymmetry in gait initiation of hemi- paretic patients as compared to healthy subjects. (2) 14 hemiparetic patients (5 men and 9 women, mean age 62.8 years), mean stroke interval 3.7 months (range, 2.1–8.3). Five patients suffered from a right and 90 from a left hemiparesis. Ten healthy subjects (5 men, 5 women, mean age 57.3 years). (3) The vertical and horizontal forces were sampled with 2 triaxial force platforms. (4) After an acoustic signal, subjects started to walk at self-adopted speed, five times each with both their right and left leg.	Longer starting cycle duration and smaller backward displacement of the CoP in hemiparetic subjects compared to healthy subjects. The medio-lateral displacement of the CoP toward the trailing leg in the initial phase was less pronounced when the patient started with the paretic leg. When the patients made the first step with the paretic leg, the resulting trajectory of the CoM in the medio-lateral plane was very similar to that of healthy subjects. AP acceleration of the CoM was found to be delayed when starting with the affected leg.
Brunt et al. (3)	(1) To explore the relation between asymmetry in leg loading of persons with stroke and their ability to generate the appropriate forces to initiate gait; and to describe the relation between EMG activity of the involved and non-involved leg and the resultant ground reaction forces. (2)13 patients with hemiplegia secondary to a cerebral vascular accident. (3) Surface electrodes were applied bilaterally to the center of the muscle belly of the medial gastrocnemius (G) and TA muscles Vertical force before gait initiation was determined on a force-plate. (4) Walking at the onset of the light signal. The leading leg was always the involved leg. Six satisfactory trials in which the first three trials began with their involved leg on the force platform and the last three trials with their uninvolved leg on the force platform.	. The greater the leading leg was loaded before movement onset the more these forces approached those values observed in healthy adults. Reduced propulsion by the paretic leg,. Correlation between symmetrical weight bearing and propulsion were observed. Decreased TA muscle activity clearly resulted in a decrease in the contribution of the leading leg to forward momentum before toe-off of that leg. There was often an increased G activity of the non-involved trailing leg that at times resulted in a TA and G co-contraction.

*Only results related to GI-APA are reported.GI-APA, anticipatory postural adjustments; COP, center of pressure; COM, center of mass; ML, medio-lateral; AP, antero-posterior; TA, tibialis anterior; G, gastrocnemius*.

### Patient Characteristics

The patients' age ranged between 21 and 86 years. In 11 of the 15 selected articles, GI was compared in healthy adults vs. stroke patients (5, 11, 21–24, 27–31). In the five remaining articles, only stroke patients were included (3, 4, 22, 25, 26). In 11 articles, participants had experienced stroke at least six months prior to GI recording (4, 5, 21, 23–26, 28–31). In one study, patients had experienced stroke 3.7 months prior to GI recording (11). In the remaining four studies, the post-stroke interval was not indicated (3, 12, 22, 27). The symptoms always consisted in hemiparesis, although its degree was rarely specified (4, 5, 25, 27–29). The presence and degree of spasticity was acknowledged only in Bensoussan et al. (4).

### Task and Recordings

In four articles, participants were asked to perform a single step in a reaction-time paradigm, i.e., after the deliverance of a GO signal (25, 27–29). In the other articles, participants initiated gait spontaneously and continued walking at their own speed. In two articles (21, 23), the contralesional leg was systematically used as the leading leg. In the other articles, both legs were used as the leading leg.

Dynamic investigation using force platforms was conducted in all articles except one (26). EMG investigation was also conducted in five articles (3, 23, 26, 27, 31).

### Quality Assessment

The results of the quality assessments for each study are given in Table 3. Only non-randomized studies corresponded to the inclusion criteria and were evaluated. According to the modified Downs and Black scale, the mean quality index score for the studies was 14 ± 1.6, with scores ranging from 11 (3) to 17 (26, 29). Only non-randomized observational studies corresponded to the inclusion criteria and were evaluated, so the level of evidence was classified as “low quality.” No treatment was administered in the studies and no meta-analysis was conducted, so no grading upwards could be done.

**Table 3:** Quality assessment analysis according to the modified Downs and Black scale.

**Authors**	**Items**																											**Total**
	**Reporting**								**External validity**				**Internal validity - bias**							**Internal validity—confounding**						**Power**		
	**1**	**2**	**3**	**4**	**5**	**6**	**7**	**8**	**9**	**10**	**11**	**12**	**13**	**14**	**15**	**16**	**17**	**18**	**19**	**20**	**21**	**22**	**23**	**24**	**25**	**26**	**27**	
Gama et al. (21)	1	1	1	1	0	1	1	0	1	1	0	0	0	0	0	1	1	1	1	1	0	0	0	0	0	1	0	14
Rajachandrakumar et al. (12)	1	1	1	1	0	1	1	0	1	1	0	0	0	0	0	1	1	1	1	1	0	0	0	0	0	1	0	14
Sharma et al. (22)	1	1	1	1	0	1	1	0	1	1	0	0	0	0	0	1	1	1	1	1	0	0	0	0	0	1	0	14
Sousa et al. (23)	1	1	1	1	0	1	1	0	1	1	0	0	0	0	0	1	1	1	1	1	0	0	0	0	0	1	0	14
Sousa et al. (24)	1	1	1	1	0	1	1	0	1	1	0	0	0	0	0	1	1	1	1	1	0	0	0	0	0	1	0	14
Martinez et al. (25)	1	1	0	1	0	1	1	0	1	1	1	1	0	0	0	1	1	1	1	1	1	0	0	0	0	1	0	16
Ko et al. (26)	1	1	1	1	0	1	1	0	1	1	1	1	0	0	0	1	1	1	1	1	1	0	0	0	0	1	0	17
Chang et al. (27)	1	1	1	1	0	1	1	0	1	1	0	0	0	0	0	1	1	1	1	1	0	0	0	0	0	1	0	14
Melzer et al. (28)	1	1	1	1	0	1	1	0	1	1	0	0	0	0	0	1	1	1	1	1	0	0	0	0	0	1	1	15
Melzer et al. (29)	1	1	1	1	0	1	1	0	1	1	1	0	0	0	0	1	1	1	1	1	1	0	0	0	0	1	1	17
Bensoussan et al. (5)	1	1	1	1	0	1	1	0	1	0	0	0	0	0	0	1	1	1	1	1	0	0	0	0	0	1	0	13
Tokuno and Eng (30)	1	1	1	1	0	1	1	0	1	0	0	0	0	0	0	1	1	1	1	1	0	0	0	0	0	1	0	13
Bensoussan et al. (4)	1	1	1	1	0	1	1	0	1	1	0	0	0	0	0	1	1	1	1	1	0	0	0	0	0	1	0	14
Kirker et al. (31)	1	1	0	1	0	1	1	0	1	0	0	0	0	0	0	1	1	1	1	1	0	0	0	0	0	1	0	12
Hesse et al. (11)	1	1	1	1	0	1	1	0	1	0	0	0	0	0	0	1	1	1	1	1	0	0	0	0	0	1	0	13
Brunt et al. (3)	1	1	0	1	0	1	0	0	1	0	0	0	0	0	0	1	1	1	1	1	0	0	0	0	0	1	0	11

*Biomechanical and EMG features of APA: Stroke vs. healthy patients*.

### Biomechanical and EMG Features of APA: Stroke vs. Healthy Patients

#### Biomechanics

##### APA pattern

Rajachandrakumar et al. (12) showed that when participants with sub-acute stroke initiated gait at their own speed, 78% exhibited at least one trial with a GI pattern classified as “atypical.” Atypical GI trial corresponded to a trial performed without APA or with “multiple” APA (i.e., with a multi-peaked CoP trace). In contrast, Martinez et al. (25) observed GI-APA in all trials that involved voluntary stepping in post-stroke patients (mean chronicity: 2.9 years), while APA were absent in 25% of trials involving perturbation-induced stepping.

##### APA duration

Rajachandrakumar et al. (12) showed that trials with multiple APA had a longer APA duration than normal GI trials (i.e., GI with a single peak CoP trace). A longer APA duration in stroke subjects compared to healthy subjects was reported by many authors (11, 23, 25, 27, 28). In addition, Gama et al. (21) recently established that stroke patients presented lower velocity of the CoP in both the ML and AP directions during the anticipatory phase of gait initiation.

##### Center-of-pressure shift

Sousa et al. (24) showed that post-stroke patients presented only half of the COP displacements (both in the frontal and sagittal plane) compared to healthy subjects. Gama et al. (21) demonstrated shorter ML and AP CoP displacements as well. A smaller COP backward shift was also demonstrated by Hesse et al. (11).

##### Ground reaction forces

Melzer et al. (28) and Sharma et al. (22) showed that anteroposterior ground reaction forces generated by both the paretic and non-paretic leg in post-stroke patients during APA were lower compared to healthy controls.

Altogether, these studies indicate that stroke patients present impaired APA compared to healthy subjects, characterized by atypical patterns, longer duration and lower amplitude. The results reported in the paragraph below show that GI parameters can also differ depending on which leg (i.e., paretic or non-paretic) is used as the leading leg.

#### Electromyography

Impairments of muscle activation during APA were demonstrated by many authors in stroke patients compared to healthy subjects (3, 23, 26, 31). Sousa et al. (24) showed that when the paretic leg was used as the leading leg, the TA of the paretic leg presented only half of the EMG activity observed in healthy subjects. On the other hand, Ko et al. (26) showed that when gait was initiated with the non-paretic leg, the TA activation in the paretic leg was increased by 27 to 36% compared with the paretic leg as leading leg. Moreover, stroke patients exhibited a bilateral decrease in TA activity during GI-APA and had difficulties in reducing tonic activity in the soleus muscle of the paretic leg (3). Finally, Kirker et al. (31) showed that when stroke patients initiated gait with the paretic leg, GI-APA were characterized by an impaired activation of the gluteus medius muscle of the paretic leg, which was compensated by more prominent activity in the contralateral adductor.

### APA in Stroke Patients: Paretic vs. Non-paretic leg as the Leading or Trailing leg

#### APA Pattern

Rajachandrakumar et al. (12) showed that the frequency of trials with no APA did not differ when gait was initiated with the paretic or non-paretic leg. In contrast, multiple APA were more prevalent when the non-paretic leg was used as the leading leg. Additionally, when stepping with the non-paretic leg, the frequency of trials with no APA was negatively correlated with motor recovery.

#### APA Duration

Bensoussan et al. (5) showed that the postural phase was longer when the paretic leg was the trailing leg. In contrast, Hesse et al. (11) and Melzer et al. (28) found no significant difference of postural phase duration when initiating gait with the paretic or non-paretic leg.

#### APA Amplitude in the Frontal Plane

Sharma et al. (22) showed that patients with left hemisphere lesions generated greater lateral ground reaction forces with the non-paretic left leg than with the paretic right leg. In contrast, medial ground reaction forces were equivalent for both the paretic and non-paretic leg. Additionally, Hesse et al. (11) reported that the amplitude of the mediolateral CoP shift during APA was lower when gait was initiated with the paretic leg. In contrast, the CoM did not move toward the trailing leg when patients started with their non-paretic leg, while the trajectory of the CoM was similar to that of healthy subjects when patients initiated gait with the paretic leg.

#### APA Amplitude in the Sagittal Plane

Regarding APA in the sagittal plane, the same authors found that anticipatory anterior-posterior CoM acceleration during APA was lower when starting with the paretic leg (22). Tokuno and Eng (30) found that the anteroposterior impulse generated by the trailing leg was less than half that of healthy subjects. Additionally, Bensoussan et al. (4, 5) found that the ground reaction forces exerted by the paretic leg were directed backwards during APA (retropulsive action) but were directed forward for the non-paretic leg (propulsive action). Altogether, these findings indicate that the use of the paretic leg as the leading leg is associated with difficulties activating the TA and gluteus medius muscles during APA, which result in reduced CoP shift and CoM acceleration. In contrast, the use of the paretic leg as the trailing leg seems to challenge balance to a greater extent during GI.

## Discussion

The aim of this systematic review was to provide an overview of the impact of stroke on the organization of APA associated with gait initiation (GI-APA). Altogether, the reviewed studies suggest that the anticipatory phase of GI is impaired in stroke patients. The findings principally highlight the changes in the pattern and spatiotemporal parameters of GI-APA in comparison with healthy subjects and the differences in initiating gait with the paretic or non-paretic leg. Moreover, this review may provide an evidenced-based theoretical framework for clinicians involved in rehabilitation programs for stroke patients.

The majority of the included studies were scored ≥ 14/28 according to the modified Downs and Black scale. The most common deficiencies in these included studies, in reference to the modified Downs and Black scale, were the lists of the principal confounders (question 5, i.e., not applicable for this review, only for interventional studies) and the adverse effects (question 8, i.e., not applicable for this review, only for interventional studies), the interventions not representative of usual care (question 13, i.e., not applicable for this review, only for interventional studies), the blinding of the patients or the assessors (questions 14 and 15, i.e., not applicable for this review, only for interventional studies), the specification of the time period of recruitment (question 22), the randomization of the subjects in groups (questions 23 and 24, i.e., not applicable for this review, only for randomized control trial studies) and the investigation of the main confounders (question 25, i.e., not applicable for this review, only for interventional studies). These absences were not surprising because the studies did not include intervention and control groups to measure the effects of treatment, but were observational studies comparing GI between subjects during a single session. Because of the absence of randomized controlled trials, a low quality level of evidence for the results may be provided (20). This means that further research with randomized protocols and more standardized protocols is very likely to have an important impact on our confidence in the results. The level of the quality assessment, according to the modified Downs and Black scale, must be considered with caution because there is no treatment group (i.e., interventional randomized controlled trials) in the included studies.

Martinez et al. (25) found that when stroke patients initiated gait, GI-APA were present in 100% of trials, whereas Rajachandrakumar et al. (12) reported the possibility of trials with no GI-APA. The subjects in these two studies were at different post-stroke times and the significant changes in motor recovery occurring in the weeks after stroke may partially explain the observed differences (32, 33). Rajachandrakumar et al. (12) found that trials with no GI-APA were more prevalent among patients who walked slowly, and that motor impairment was correlated with the frequency of trials with no GI-APA. Multiple GI-APA, the other atypical pattern described by the authors, was observed at a higher frequency when gait was initiated with the healthy leg.

The “psychological apprehension” loading of the paretic leg may partially explain longer GI-APA duration and may reflect the priority of the central nervous system to secure mediolateral stability before walking (4, 5, 12). A longer GI-APA duration can also be attributed to delays in soleus deactivation (23) and TA activation (27). These muscle impairments probably lead to difficulties in correctly timing the CoP and CoM shift during GI-APA compared to healthy subjects.

Lower GI-APA amplitude observed in both the frontal and sagittal planes may result in lower stability and lower progression velocity, respectively, during GI (11, 21–23, 28, 30). However, the hypothesis that the lower gait progression velocity observed in stroke patients results from GI-APA impairments requires further investigation (23). Indeed, it has been shown that the amplitude of COP displacement is similar between healthy and post-stroke participants when healthy participants are instructed to walk at a speed similar to that chosen by the post-stroke subjects (30). Thus, the lower GI-APA amplitude reported in stroke patients under spontaneous velocity conditions appears to result from their lower self-adopted speed compared to healthy participants. Furthermore, balance and performance during the execution phase is affected by the choice of the leading leg during the postural phase. GI-APA are dynamic phenomena that could impact balance (34–36) and an inability to support body weight during GI due to weakness in the paretic leg. Thus, lower GI-APA amplitude in stroke patients could result from postural muscle weakness or reflect adaptations to the lower balance state.

Many studies included in the present review confirm the findings of Pélissier et al. (37) who described a reduction in the duration of the swing phase, step length, walking velocity, and magnitude of the propulsion force during the execution phase when initiating gait with the healthy leg. These effects could be the result of decreased plantar flexor, hip flexor and hip extensor strength on the paretic side (38, 39), which may underlie the inability to support body weight. The incapacity to correctly propel the CoM above the paretic leg during the stance phase may explain the higher degree of instability observed when starting with the non-paretic leg (3–5, 11, 22, 30, 31). Additionally, the paretic leg muscles contribute less to the load of the paretic side, generate less power, and may even produce retropulsive forces, which may reduce walking velocity, because all of the propulsive forces are generated by the healthy leg alone (4, 5, 40).

Knowing the main characteristics of postural deficiencies during gait initiation or steady walking in stroke patients may help clinicians assess the efficiency of neurorehabilitation methods and provide an evidence-based theoretical framework for motor recovery. For example, the review of (37) established that the symmetry of stride was not correlated with walking performance (especially with the progression velocity). In addition, partial body support (i.e., on the non-paretic stance leg) was shown to have no effect on the anticipatory phase (21). These findings suggest that it is not necessary to obtain symmetrical static balance before starting gait rehabilitation. On the contrary, gait training should be started as early as possible after stroke. As documented in the literature reviewed above, initiating gait with the paretic leg improves stability and motor performance, so we recommend that rehabilitation programs promote this strategy in the early post-stroke period to improve patient mobility and autonomy.

Gamma et al. (21) recommend the use of an over-ground body weight support to improve mediolateral APA (i.e., stability) without changing the anteroposterior APA velocity (i.e., performance). However, in practice the combination of different rehabilitation strategies, notably the use of functional electrical stimulation and brain-computer interfaces, seems to be more effective than over-ground gait training alone (41).

Moreover, since using the paretic leg as the trailing leg has been found to increase the amplitude of TA muscle activity by between 27 and 36% (26), this strategy can be used as TA strength training to improve postural muscle performance in the paretic leg. Regarding possible gait-rehabilitation strategies, increasing TA muscle activity of the paretic leg during gait initiation seems to be an important target for motor rehabilitation, since bilateral TA activation during APA serves to provide initial propulsive forces that shift the whole-body forward (3). It is also involved in body balance maintenance during step execution (10). It is clear that strength training should not be restricted to the TA of the paretic limb but should involve all paretic leg muscles. Specifically, reinforcing hip abductor muscles may facilitate the anticipatory CoP and CoM shifts in the frontal plane (31), while reinforcing hip extensors, knee extensors and plantar flexors should facilitate body weight support against gravity and provide efficient propulsive forces in the sagittal plane (5, 22, 28, 30).

In this way, the use of virtual reality training during gait initiation could help train and obtain recovery of force and power separately for each lower limb (i.e., paretic and non-paretic limb) in individuals with stroke (42).

## Conclusion

This systematic review provides an update on GI-APA reorganization following stroke. Stroke patients present atypical GI-APA patterns, longer GI-APA duration and lower GI-APA amplitude compared to healthy people, regardless of which leg is used as the leading or trailing leg. GI is facilitated when the non-paretic leg is used as the trailing leg because the weakness of the paretic leg leads to difficulties in supporting body weight during the upcoming stance phase. Further experiments should include distinct groups of patients in order to describe GI-APA features in acute, subacute and chronic stroke, and the influence of spasticity and of the lesion site. Understanding the changes in each population could be relevant for personalizing rehabilitation strategies.

## Author Contributions

AD wrote the manuscript. TV and TH performed the bibliographical search and selected the articles to be included in the review. EY, J-LH, and SC reviewed the manuscript.

### Conflict of Interest Statement

The authors declare that the research was conducted in the absence of any commercial or financial relationships that could be construed as a potential conflict of interest.
